# Effect of External Surface Diffusion Barriers on Platinum/Beta‐Catalyzed Isomerization of *n*‐Pentane

**DOI:** 10.1002/anie.202104859

**Published:** 2021-05-17

**Authors:** Shen Hu, Junru Liu, Guanghua Ye, Xinggui Zhou, Marc‐Olivier Coppens, Weikang Yuan

**Affiliations:** ^1^ State Key Laboratory of Chemical Engineering School of Chemical Engineering East China University of Science and Technology Shanghai 200237 China; ^2^ Department of Chemical Engineering University College London London WC1E 7JE UK

**Keywords:** diffusion, isomerization, surface barriers, surface modification, zeolite Beta

## Abstract

We have developed a generalizable strategy to quantify the effect of surface barriers on zeolite catalysis. Isomerization of n‐pentane, catalyzed by Pt/Beta, is taken as a model reaction system. Firstly, the surface modification by chemical liquid deposition of SiO_2_ was carried out to control the surface barriers on zeolite Beta crystals. The deposition of SiO_2_ leads to a very slight change in the physical properties of Beta crystals, but an obvious reduction in Brønsted acid sites. Diffusion measurements by the zero‐length column (ZLC) method show that the apparent diffusivity of n‐pentane can be more than doubled after SiO_2_ deposition, indicating that the surface barriers have been weakened. Catalytic performance was tested in a fixed‐bed reactor, showing that the apparent catalytic activity improved by 51–131 % after SiO_2_ deposition. These results provide direct proof that reducing surface barriers can be an effective route to improve zeolite catalyst performance deteriorated by transport limitations.

## Graphical abstract

External surface diffusion barriers on zeolite Beta crystals significantly reduce their apparent catalytic activity in catalyzing isomerization of *n*‐pentane. Eliminating surface barriers can lead to an improvement in apparent catalytic activity higher than 131 %.

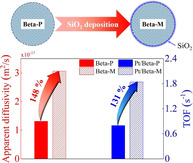

Zeolites are an important class of microporous materials that are widely used as catalysts in the refining and petrochemical industries. Due to their well‐defined, molecular‐sized micropore networks, they possess the great advantage of shape selectivity in catalysis,^[1]^ but they are also heavily plagued by diffusion limitations that reduce catalytic activity, selectivity, and stability.^[2]^ Great efforts have been devoted to mitigate diffusion limitations in zeolite crystals by synthesizing hierarchically structured zeolites with shortened diffusion path lengths in micropores.[^3^, ^4^] However, recent observations show that diffusion limitations still persist in zeolite crystals, even when their thickness is reduced to a few nanometers.[^5^, ^6^] In this case, the external surface diffusion barriers should be the dominant mass transfer resistance.

For decades, surface barriers were speculated to be present on zeolite crystals, as a difference of several orders of magnitude in apparent diffusivity could be measured for zeolite crystals of different sizes.[^6^, ^7^, ^8^, ^9^, ^10^] Kärger and co‐workers[^11^, ^12^, ^13^, ^14^, ^15^] observed the existence of surface barriers using unique micro‐imaging methods (e.g., interference microscopy and IR microscopy) and pulsed‐field gradient nuclear magnetic resonance (PFG NMR) spectroscopy. They found that the concentration of a probe molecule close to the outer boundary of the zeolite crystals shows a jump in comparison with the equilibrium value finally attained with the progression of adsorption or desorption, which could only be explained when accounting for the presence of surface barriers. Surface barriers may originate from surface defects (e.g., narrowing, blocking, and misalignment), which is supported by experimental and computational studies,[^8^, ^16^, ^17^, ^18^, ^19^, ^20^] while some computational works[^21^, ^22^, ^23^, ^24^, ^25^] show that a perfect, simulated zeolite crystal surface can also result in surface barriers.

Although these acknowledgements of surface barriers on zeolite crystals are obtained from observing sorption processes, they suggest that surface barriers should also play an important role in affecting catalysis in zeolite crystals. Understanding this role may elicit a new route to improve zeolite catalysts. However, little attention has been paid to this research topic, except for recent works by Rao et al.^[26]^ and Peng et al.^[27]^ Rao et al.^[26]^ found that their model can predict the experimental data for ZSM‐5‐catalyzed benzene alkylation with ethylene only when surface barriers are included. Peng et al.^[27]^ reported that SAPO‐34 surface‐modified by acid etching shows longer catalyst lifetime and higher selectivity in catalyzing MTO reactions, and they related these improvements to the increased surface permeability after the acid etching. Up to now, we still lack adequate knowledge about how to control surface barriers in zeolite catalysis.

To investigate the effect of surface barriers on zeolite catalysis, a proper strategy needs to be developed. Due to the presence of potential surface reactions, it is almost impossible to measure surface barriers under reaction conditions.^[28]^ In this work, we developed a strategy: (1) SiO_2_ deposition on a parent zeolite crystal to regulate surface barriers; (2) diffusion measurements of the modified and parent zeolites to reveal the effect of surface barriers on diffusion; (3) reaction tests of the two zeolites to quantify the effect of surface barriers on the apparent catalytic activity. SiO_2_ deposition has been proven to be an effective way to regulate surface barriers in studies on adsorption in zeolites.[^29^, ^30^] The zero‐length column (ZLC) method is employed to measure diffusion, since this method is accurate, yet efficient. Isomerization of *n*‐pentane catalyzed by Pt/Beta is taken as the model reaction system, as there exist strong diffusion limitations and regulating surface barriers may yield a remarkable influence on the catalytic performance.^[31]^ It should be noted that the elementary reactions on Pt sites reach equilibrium very quickly and the reactions on Brønsted acid sites normally determine the intrinsic reaction rate,^[32]^ which is also proven in Figure S6; further experimental details are given in the Supporting Information.

Figure 1 displays the physical properties of the parent zeolite Beta (Beta‐P) and the SiO_2_‐modified zeolite Beta (Beta‐M), using the chemical liquid deposition (CLD) method. The XRD patterns of the two samples display peaks at 2*θ*=7.8°, 13.4°, 21.5°, 22.6°, 25.4°, 27.0°, and 29.7° attributed to the typical Beta phase with a BEA‐type structure,^[33]^ indicating that SiO_2_ deposition does not change the framework structure of Beta. The peak at 22.6° is slightly broadened after SiO_2_ deposition, which can be attributed to the presence of an amorphous phase on the zeolite Beta crystal.^[34]^ As seen from the SEM images, both samples possess well‐faceted external shapes with an average particle size around 440 nm, and no observable changes in morphology are generated after SiO_2_ deposition. HRTEM images also show no observable differences in surface structure between Beta‐P and Beta‐M. Besides, their SAED patterns display bright spots, suggesting that both Beta‐P and Beta‐M are single‐crystalline. This observation excludes the potential effect of internal diffusion barriers.^[35]^ Both samples exhibit a type I isotherm according to the IUPAC classification, which is typical for microporous materials. The isotherm of Beta‐M shows a slightly higher uptake at *p*/*p* ^0^=0.99, which agrees with the presence of a small quantity of amorphous phase after SiO_2_ deposition.

**Figure 1:**
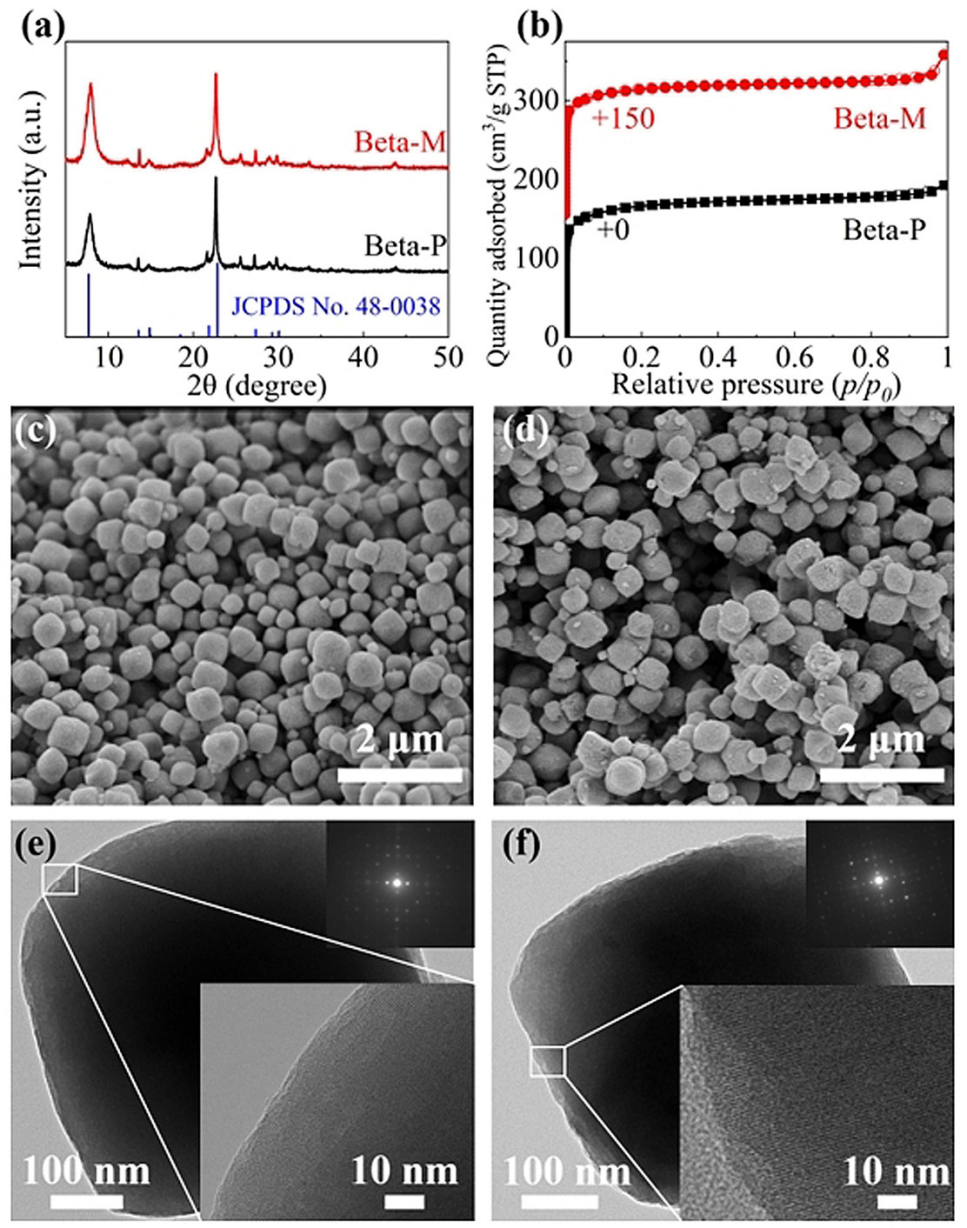
Structure, morphology, and texture of Beta‐P and Beta‐M. a) Wide‐angle powder XRD patterns; b) N_2_ adsorption and desorption isotherms; representative SEM images of c) Beta‐P and d) Beta‐M; representative HRTEM images of e) Beta‐P and f) Beta‐M, where the inserts with a black background are SAED patterns.

XRD patterns and adsorption isotherms indicate that the external surface of the Beta crystals might indeed have been modified by amorphous SiO_2_ after performing the chemical liquid deposition; this is not obvious, and the surface modification is also too slight to be observed in SEM and HRTEM images. However, the measurement of Si/Al molar ratios gives further evidence, as shown in Table 1. The Si/Al ratios measured using ICP‐AES and EDS reflect the fractions of Si in the whole sample and near the exterior of the crystals, respectively. The Si/Al ratios for Beta‐M are higher than those for Beta‐P, especially for the Si/Al ratios measured using EDS. More evidence of surface modification by SiO_2_ can also be found in Figure 2.

**Figure 2:**
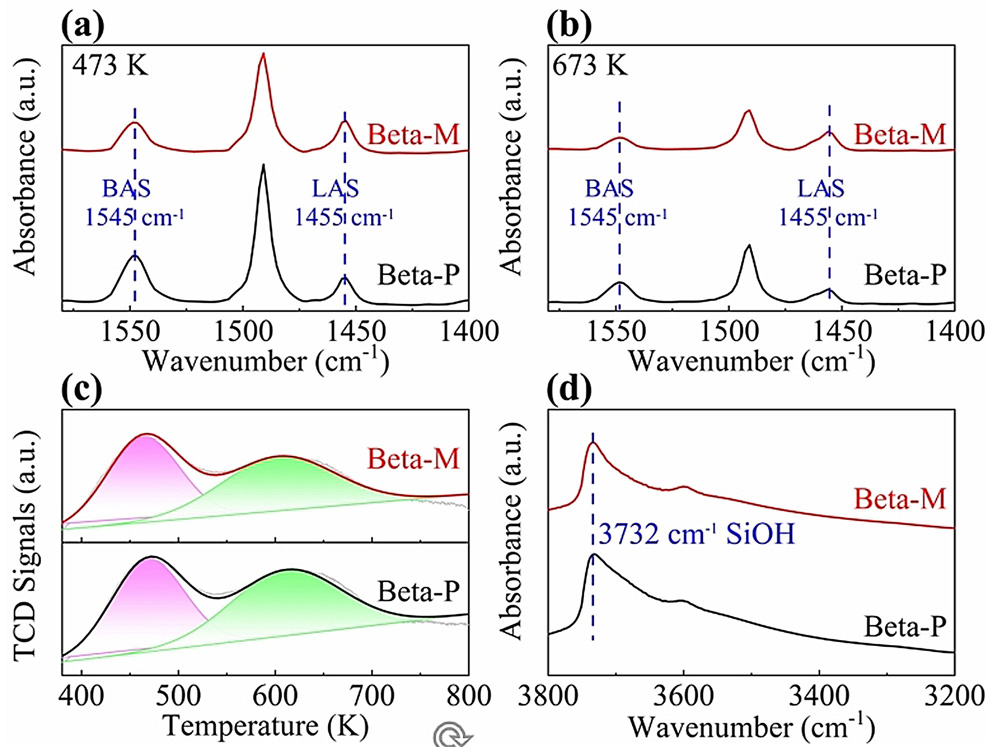
Acidity of Beta‐P and Beta‐M. Py‐IR curves at a) 473 K and b) 673 K; c) NH_3_‐TPD profiles; d) FT‐IR curves.

**Table 1:** Some characteristics of Beta‐P and Beta‐M. More characteristics are summarized in Table S1 of the Supporting Information.

Samples	S_BET_^[a]^ [m^2^ g^−1^]	*V*_tot_^[b]^ [cm^3^ g^−1^]	Si/Al^[c]^	Si/Al^[d]^	H^+^‐473 K^[e]^ [mmol g^−1^]	H^+^‐673 K^[e]^ [mmol g^−1^]
Beta‐P	514	0.30	25.1	28.8	0.132	0.045
Beta‐M	505	0.32	26.6	33.6	0.093	0.032

[a] Specific surface area calculated by the BET method. [b] Total pore volume determined from the adsorbed volume at *p*/*p*_0_=0.99. [c] Si/Al molar ratio measured using ICP‐AES. [d] Si/Al molar ratio measured using EDS. [e] Number of Brønsted acid sites measured from Py‐IR spectra at 473 K (H^+^‐473K) and 673 K (H^+^‐673K).

Figure 2 shows the acidic properties of Beta‐P and Beta‐M, obtained via pyridine IR spectroscopy (Py‐IR) and ammonia temperature‐programmed desorption (NH_3_‐TPD). Py‐IR spectra show bands at 1545 cm^−1^, 1455 cm^−1^, and 1490 cm^−1^, corresponding to Brønsted acid sites (BAS), Lewis acid sites (LAS), and the interaction of pyridine with both BAS and LAS.^[36]^ The total number of BAS (H^+^‐473K) decreases by 30 % after SiO_2_ deposition, while that of LAS (L‐473K) increases by 41 %. The number of strong BAS (H^+^‐673K) decreases by 29 % after SiO_2_ deposition, and that of LAS (L‐673K) increases by 37 %. BAS are believed to be the active sites for the isomerization of *n*‐alkanes,^[37]^ and their numbers are listed in Table 1. More acidic properties are collected in Table S1. The significant reduction in BAS number after SiO_2_ deposition has been extensively reported in the literature; Table S4 gives some examples.

During the chemical liquid deposition of tetraethoxysilane (TEOS), TEOS can react with bridging hydroxy groups (SiAlOH) primarily located in the pore mouth region, as TEOS (approx. 10.3 Å) is larger than the pore openings of zeolite Beta (7.6 Å×6.4 Å).^[38]^ This silylation reaction reduces the number of BAS. Meanwhile, TEOS can also passivate silanol groups (SiOH) on the zeolite Beta crystal,^[39]^ as indicated by the reduced intensity of the FTIR peak at 3732 cm^−1^ (see Figure 2 d). It should be noted that dealumination may occur during chemical liquid deposition, turning a small amount of framework Al species (FAl) into extra‐framework Al species (EFAl).^[40]^ This may also lead to some of the decrease of BAS and explain the increase of LAS after SiO_2_ deposition (see Table S1).

The apparent diffusivities of a probe molecule (i.e., *n*‐pentane) in Beta‐P and Beta‐M were measured using the ZLC method to reveal how SiO_2_ deposition affects surface barriers in this case. Figure 3 shows the desorption curves and the apparent diffusivities fitted from these curves. After SiO_2_ deposition, the time for reducing *C*/*C*_0_ to 0.001 is shortened significantly and the apparent diffusivity is increased by 121–148 %. As SiO_2_ deposition does not change the interior structure of the Beta crystals, so that the intracrystalline diffusivities in the two samples should be the same,^[27]^ this increase in apparent diffusivity can be purely attributed to the reduction of surface barriers after SiO_2_ deposition, since only the crystal surface is modified. The apparent activation energies of transport in Beta‐P and Beta‐M are close, while the pre‐exponential factor for Beta‐M is 265 % larger (see Table S6). This implies that SiO_2_ deposition may increase the sticking probability of molecules on zeolite Beta.[^30^, ^41^]

**Figure 3:**
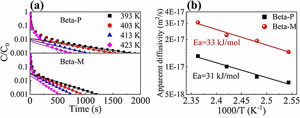
Apparent diffusivities of *n*‐pentane measured by the zero‐length column method. a) Desorption curves (*C*_0_ and *C* are the initial and transient effluent concentrations of *n*‐pentane); b) Arrhenius plots of apparent diffusivities. Measuring conditions: Flow rate=80 mL min^−1^, atmospheric pressure.

The catalytic performance of Pt/Beta‐P and Pt/Beta‐M was tested in a fixed‐bed reactor to quantify the effect of surface barriers on isomerization of *n*‐pentane. Both samples show high selectivity (>95 %) and stability at reaction temperatures of 573–643 K, and the slight decrease in conversion after 15 h on stream can be attributed to the deactivation by coking.^[42]^ At 573 K, the conversion for Pt/Beta‐M at *t*=1 h is 15 % lower than for Pt/Beta‐P, as diffusion limitations at this temperature are so slight that the conversion is primarily determined by the BAS number. With increased reaction temperature, diffusion limitations become important. At 593 and 613 K, the conversions for Pt/Beta‐M at *t*=1 h are 22 % and 31 % higher than those for Pt/Beta‐P (so the order of observed activity is inverted), since SiO_2_ deposition can reduce surface barriers and, thus, the interior of Beta‐M crystals is more accessible. When the temperature is too high (>633 K), the diffusion limitations are so strong that reactions primarily occur in a very thin surface layer of the Beta crystal. Meanwhile, SiO_2_ deposition can significantly passivate BAS located in the surface region. In this case, with the increase in reaction temperature, the improvement in conversion becomes less and less significant after SiO_2_ deposition, and the conversion for Pt/Beta‐M can even be lower than for Pt/Beta‐P, as seen from Figure 4 d,e.

**Figure 4:**
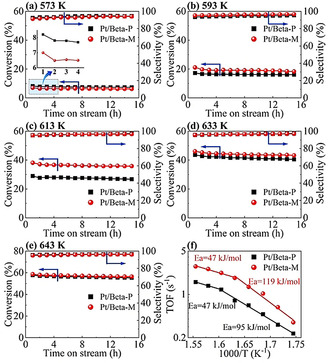
Catalytic performance of Pt/Beta‐P and Pt/Beta‐M. Conversion of *n*‐pentane and selectivity to *i*‐pentane at temperatures of a) 573 K, b) 593 K, c) 613 K, d) 633 K, e) 643 K. f) Arrhenius plots of the TOFs at *t*=1 h. Reaction conditions: *P*=1 atm, WHSV=45.6 g_*n*‐pentane_ g_Cat_^−1^ h^−1^, H_2_/*n*‐pentane mole ratio=15.8.

To compare the apparent activities of Pt/Beta‐P and Pt/Beta‐M on the same basis, the turnover frequencies (TOFs) based on the total BAS number (H^+^‐473K) are calculated and compared in Arrhenius plots (see Figure 4 f). In the temperature range of 573–643 K, the TOF for Pt/Beta‐M is 51–131 % higher than for Pt/Beta‐P. This indicates that eliminating surface barriers can lead to an improvement in apparent catalytic activity higher than 131 % in this reaction system. At low temperatures, the apparent activation energy for Pt/Beta‐M is 119 kJ mol^−1^, which is close to the one (124 kJ mol^−1^) measured by Matsuda et al.^[43]^ under the conditions where diffusion limitations are negligible; the one for Pt/Beta‐P is 95 kJ mol^−1^, indicating that diffusion resistances start to play a role and surface barriers contribute a lot to these resistances. At high temperatures, the apparent activation energies for both Pt/Beta‐M and Pt/Beta‐P become 47 kJ mol^−1^, suggesting the existence of significant diffusion limitations.^[44]^

The effect of increased SiO_2_ loading on catalytic performance has also been probed in this work, and these results are given in the Supporting Information. The results show that the conversion at *t*=1 h for Pt/Beta‐M‐M (27.7 wt % SiO_2_) is 3.8–19.3 % lower than for Pt/Beta‐P. Too much SiO_2_ on Beta crystals may block many surface pores and significantly passivates active sites. Thus, an optimal SiO_2_ loading is anticipated, but obtaining this optimal loading is out of the scope of this article and will be the subject of future work.

In conclusion, we have developed a strategy combining surface modification, diffusion measurements, and catalytic tests, in order to quantify the effect of external surface diffusion barriers on zeolite catalysis. In this work, SiO_2_ deposition by chemical liquid deposition was used to modify the surface of zeolite Beta and, subsequently, to regulate surface barriers; then, the apparent diffusivity of the reactant (i.e., *n*‐pentane) was measured using the ZLC method to reveal how SiO_2_ deposition affects surface barriers; lastly, the catalytic performance for isomerization of *n*‐pentane was tested in a fixed‐bed reactor to quantify the influence of surface barriers on zeolite catalysis. The results show that SiO_2_ deposition can only lead to a very slight change in textural properties, but an obvious reduction in BAS number. After the surface modification, surface barriers are significantly reduced, leading to a 121–148 % increase in the apparent diffusivity of *n*‐pentane and a 51–131 % improvement in the apparent catalytic activity. This work provides a new strategy to investigate and control the role of surface barriers in zeolite catalysis, and the results suggest that surface barriers should be accounted for when developing zeolite catalysts used in industry.

## Conflict of interest

The authors declare no conflict of interest.

## Supporting information

As a service to our authors and readers, this journal provides supporting information supplied by the authors. Such materials are peer reviewed and may be re‐organized for online delivery, but are not copy‐edited or typeset. Technical support issues arising from supporting information (other than missing files) should be addressed to the authors.

Supplementary
