# Supplementary material for: Effect of External Surface Diffusion Barriers on Platinum/Beta‐Catalyzed Isomerization of n‐Pentane
Source: Angew Chem Int Ed Engl. 2021 May 17;60(26):14394–8. doi: 10.1002/anie.202104859 (PMC8252482; doi:10.1002/anie.202104859)
Supplement: Supplementary file 1 — Supplementary [file ANIE-60-14394-s001.pdf]

Angewandte Chemie
Eine Zeitschrift der Gesellschaft Deutscher Chemiker

Supporting Information

Effect of External Surface Diffusion Barriers on Platinum/Beta-Catalyzed Isomerization of *n*-Pentane

Shen Hu, Junru Liu, Guanghua Ye, Xinggui Zhou, Marc-Olivier Coppens,* and Weikang Yuan*

anie_202104859_sm_miscellaneous_information.pdf

Table of Contents

1. Experimental Procedures......S3

1.1. Catalyst preparation......S3

1.2. Catalyst characterization......S4

1.3. Catalytic tests......S6

1.4. Zero length column (ZLC) measurements......S7

2. Results and Discussion......S9

2.1. Characteristics of Beta samples......S9

2.2. Pore size distributions of Beta samples......S10

2.3. HRTEM images and EDS-Mapping images......S11

2.4. Particle size distributions of Beta samples......S12

2.5. Pt size distributions of Pt/Beta samples......S13

2.6. Elimination of the effect of Pt......S14

2.7. Effect of SiO_2 deposition on acidity......S16

2.8. ZLC results......S17

2.9. Effect of SiO_2 loading......S18

References......S23

Author Contributions......S24

1. Experimental Procedures

1.1. Catalyst preparation

Synthesis of zeolite Beta

Zeolite Beta was synthesized as follows, based on our previous work.[1] Appropriate amounts of sodium hydroxide (≥98%, Shanghai Titan Scientific Co. Ltd), deionized water, and tetraethylammonium hydroxide (TEAOH) (25 wt% in water, Energy Chemical) were mixed and then stirred for half an hour to get solution A. Then, silica sol (40 wt% suspension in water, Sigma-Aldrich) was added into solution A, and the mixture was stirred for 4 hours to obtain solution B. Dissolved sodium aluminate (Al_2O_3≥41%, Shanghai Titan Scientific Co. Ltd) was added dropwise into solution B, and the mixture was stirred for another 3 hours. The molecular composition of the final mixture was $1SiO_2$:0.31TEAOH:0.022NaOH:$0.021Al_2O_3$:$15.2H_2O$, and this mixture was hydrothermally treated in a Teflon-lined stainless steel autoclave at 393 K for 8 days. After the hydrothermal treatment, the product was washed with deionized water and centrifuged for three times, and then dried at 373 K overnight. Finally, the product was calcined in a muffle furnace at 823 K for 6 hours with a temperature ramp of 1.5 K/min.

To obtain the protonated form of zeolite Beta, the calcined product was ion-exchanged with a solution of 1 M NH_4Cl (≥99.5%, Shanghai Titan Scientific Co. Ltd) at 353 K for 8 hours followed by centrifugation and drying at 383 K overnight, and then the sample was calcined at 723 K for 3 hours. The ion exchange process was repeated three times, and the obtained product was labeled as Beta-P.

Chemical liquid deposition of SiO_2

Beta-P was modified by the chemical liquid deposition (CLD) method with tetraethyl orthosilicate (TEOS) (98%, Shanghai Macklin Biochemical Co., Ltd), based on the work by Reitmeier *et al.*[2] In a typical deposition process, 2 g Beta-P zeolite was dispersed in the mixture of 50 mL ethanol (⩾99.8%, LABOR) and 0.3 mL TEOS. Then, the suspension was heated and refluxed at 333 K for 1 hour. Thereafter,

ethanol was removed by evaporation in a fume hood overnight. Finally, the product was calcined at 823 K for 6 hours with a temperature ramp of 1.5 K/min. The obtained product was labeled as Beta-M.

To investigate the effects of the quantity of SiO_2 on surface barriers and catalytic activity, we prepared another SiO_2-modified sample (Beta-M-M) with a higher loading of SiO_2 than Beta-M. The aforementioned procedure for SiO_2 deposition was repeated five times to obtain Beta-M-M.

Platinum loading

Both Beta-P and Beta-M were loaded with 0.5 wt% Pt by incipient wetness impregnation with a solution of H_2PtCl_6 (Pt⩾37%, Shanghai Aladdin Bio-Chem Technology Co., LTD). Thereafter, the samples were aged overnight, followed by calcination at 723 K for 3 hours with a temperature ramp of 1.5 K/min. The obtained catalysts were labeled as Pt/Beta-P and Pt/Beta-M, respectively. To investigate the effect of Pt on isomerization of *n*-pentane, a commercial zeolite Beta (NKC Co., Ltd) was loaded with 0.3 wt% Pt and 0.5 wt% Pt, and the obtained catalysts were labeled as 0.3 wt% Pt/Beta-C and 0.5 wt% Pt/Beta-C, respectively.

1.2. Catalyst characterization

XRD

X-ray diffraction (XRD) patterns were recorded using a D8 Advance A25 diffractometer (Bruker, Germany) equipped with a Cu Kα radiation source, and the measurements were conducted in the range of $5° < 2\theta < 50°$ at a rate of 10°/min.

N_2 sorption

N_2 adsorption and desorption isotherms were measured using an ASAP 2020 instrument (Micromeritics, USA) at 77 K. The total pore volume was determined at a relative pressure of p/p_0 = 0.99. The surface area and pore size distribution were calculated using the Brunauer−Emmett−Teller (BET) method, and the Non-Local Density Functional Theory (NLDFT) method, respectively.

SEM

Scanning electron microscopy (SEM) images were taken with a NOVA Nano SEM450 microscope (FEI, USA) operating at 3 kV.

HRTEM

High-resolution transmission electron microscopy (HRTEM) and selected area electron diffraction (SAED) were performed on a JEM 2100 instrument (JOEL, Japan) operating at 200 kV.

HAADF-STEM

High-angle annular dark-field scanning transmission electron microscopy (HAADF-STEM) images were obtained using a Tecnai G2 F20 S-Twin equipped with a digitally processed STEM imaging system.

ICP-AES

Chemical compositions of Beta-P and Beta-M were analyzed by inductively coupled plasma-atomic emission spectroscopy (ICP-AES), using an IRIS 1000 instrument (Thermal Elemental, USA).

FTIR

Fourier transform infrared (FTIR) spectra were collected in the range of 400–4000 cm^{-1} with a resolution of 4 cm^{-1} using a magna-IR550 (Nicolet Company, USA).

Py-IR

The acidic properties were obtained from pyridine-adsorbed infrared (Py-IR) spectra using a Tensor 27 spectrometer (Bruker, Germany). After being pressed into a self-supporting wafer, the sample was preheated to 723 K under vacuum (1.3×10^{-2} Pa) and maintained at this temperature for half an hour. The number of Brønsted and Lewis acid sites was calculated from the amount of adsorbed probe molecules, on the basis of the areas of the characteristic bands and the associated molar extinction coefficients ($\varepsilon_{Brønsted}$ (1545 cm^{-1}) = 1.67 cm μmol^{-1}, ε_{Lewis} (1455 cm^{-1}) = 2.22 cm μmol^{-1}).[3]

NH_3-TPD

Temperature programmed desorption of ammonia (NH_3-TPD) was conducted with an Auto Chem II 2920 instrument (Micromeritics, USA). The sample was activated at 723 K in Ar flow (30 mL/min) for 1 hour. Prior to the desorption, the sample was kept at 320 K in NH_3 flow (30 mL/min) for half an hour,

followed by the purge of Ar flow (30 mL/min) for 1 hour at 373 K. The thermal conductivity detector (TCD) signals were recorded when heating the sample from 373 K to 923 K with a temperature ramp of 10 K/min in He flow (30 mL/min).

CO-Chemisorption

The dispersion of Pt was determined by CO-Chem using an Auto Chem II 2920 instrument (Micromeritics, USA). Firstly, 0.1 g catalyst was reduced at 723 K in 10% H_2/Ar flow (30 mL/min) for 2 hours. After reduction, the catalyst was purged with Ar flow (30 mL/min), before being cooled down to 320 K. Then, a CO pulse was introduced over the reduced catalyst and the CO uptake for each pulse was detected.

1.3. Catalytic tests

The performance of the materials in catalyzing *n*-pentane isomerization was tested in a fixed bed reactor under different temperatures. The fixed bed reactor was equipped with a quartz tube with an inner diameter of 7 mm. In a typical catalytic test, 0.02 g catalyst was well dispersed with 0.18 g silica sand and, then, this mixture was loaded into the quartz tube. Before the catalytic reaction, the catalyst was reduced at 723 K in H_2 flow (30 mL/min) for 2 hours with a temperature ramp of 3.5 K/min; then, the catalyst was cooled down to the reaction temperature in H_2 flow. Subsequently, *n*-pentane (>99 wt%, Alfa Aesar) was introduced into the reactor by bubbling H_2 in a saturator. The saturator was loaded with liquid *n*-pentane and kept at 273.4 K, yielding a flow of binary mixture of H_2 and *n*-pentane in which the partial pressure of *n*-pentane is 0.24 atm. The H_2 flow rate was adjusted to keep $n(H_2)$: n(n-pentane) = 15.8. The weight hourly space velocity (WHSV) of *n*-pentane was 45.6 kg *n*-pentane/(kg_{Cat}·h). The products were analyzed using an on-line gas chromatograph (FuLi GC9790II, China), equipped with a flame ionization detector (FID) and a HP-PONA capillary column (50 m×0.2 mm×0.5 mm).

1.4. Zero length column (ZLC) measurements

The apparent diffusivities of *n*-pentane in Beta-P and Beta-M were determined using the ZLC method developed by Eic and Ruthven.[4] The experiments were carried out under different temperatures to determine the activation energy of the apparent diffusivity. Prior to the measurement, the sample was activated in He flow (80 mL/min) at 473 K overnight. Then, the probe molecule (*n*-pentane) was introduced into the sample cell by bubbling He in a saturator loaded with liquid *n*-pentane and kept at 264.4 K. After the adsorption of *n*-pentane on the sample reached equilibrium, the gas flow was switched to purge the sample with He, and the transient effluent concentration was measured by a flame ionization detector (FID). The apparent diffusivity (D_{app}) could be obtained using a long-time analysis, from the desorption curve described by:

$$\frac{C}{C_0} = 2L\sum_{n=1}^{\infty}\frac{\exp(-\beta_n^2\frac{D_{app}}{R^2}t)}{[\beta_n^2 + L(L-1)]} \quad (1),$$

where C_0 and C are the initial and transient effluent concentrations of the probe molecule, here *n*-pentane, R is the radius of zeolite sample, and t is the time. β_n in Eq. (1) can be obtained from:

$$\beta_n \cot(\beta_n) + L - 1 = 0 \quad (2),$$

where L is:

$$L = \frac{1}{3}\frac{FR^2}{KV_S D_{app}} \quad (3),$$

where F is the flow rate of the purge gas, K is the Henry constant, and V_s is the volume of the sample cell. In the long-time regime, Eq. (1) can be reduced to:

$$\frac{c}{c_0} = \frac{2L}{\beta_1^2 + L(L-1)}\exp(-\beta_1^2\frac{D_{app}}{R^2}t) \quad (4),$$

where D_{app}/R^2 can be obtained by plotting $\ln(c/c_0)$ vs. t.

The apparent diffusivity measured by the ZLC method includes the effect of intracrystalline diffusion and surface barriers. The relation between the apparent diffusivity (D_{eff}), intracrystalline diffusivity (D), and surface permeability (α) can be described by:

$$\frac{1}{D_{eff}} = \frac{1}{D} + \frac{3}{\alpha l} \quad (5),$$

where l is the half thickness of a plane zeolite sheet. According to the work by Gao et al.,[5] for a spherical zeolite crystal with a radius of R, l can be calculated by:

$$l = \frac{R}{\sqrt{5}} \quad (6).$$

To calculate the surface permeability, we made two assumptions: (1) the intracrystalline diffusivities in Beta-P and Beta-M are the same; (2) the surface barriers for Beta-M are negligible. The first assumption has been well accepted, and experimental evidence for this was given by Peng et al.[6] The second assumption may not yield a quantitatively correct surface permeability, but the surface permeability should be qualitatively insightful. In addition to surface permeability, additional path length (σ) is also widely used to quantify the effect of surface barriers, and it can be calculated by using the following equation obtained from the literature:[7]

$$\tau_{eff} = \frac{R^2}{D_{eff}} = \frac{(R+\sigma)^2}{D} \quad (7),$$

where τ_{eff} is the characteristic time for diffusion. The surface permeabilities and additional path lengths for Beta-P are summarized in Table S7.

2. Results and Discussion

2.1. Characteristics of Beta samples

Table S1. Characteristics of Beta-P and Beta-M in this work.

Properties	Unit	Beta-P	Beta-M
Si/Al ratio [a]	/	25.1	26.6
Si/Al ratio [b]	/	28.8	33.6
S_{BET} [c]	m^2/g	514	505
V_{total} [d]	cm^3/g	0.30	0.32
V_{micro} [e]	cm^3/g	0.24	0.24
NH_3-W [f]	mmol/g	0.070	0.066
NH_3-S [g]	mmol/g	0.078	0.060
H^+-473 K [h]	mmol/g	0.132	0.093
L-473 K [i]	mmol/g	0.051	0.072
H^+-673 K [j]	mmol/g	0.045	0.032
L-673 K [k]	mmol/g	0.038	0.052

[a] Si/Al molar ratio measured by using ICP-AES.
[b] Si/Al molar ratio measured by using EDS.
[c] Specific surface area calculated by the BET method.
[d] Total volume determined from the adsorbed volume at p/p^0 = 0.99.
[e] Micropore volume calculated by the t-plot method.
[f,g] Numbers of weak (NH_3-W) and strong (NH_3-S) acid sites calculated from NH_3-TPD.
[h,i,j,k] Numbers of Brønsted (H^+) and Lewis (L) acid sites calculated from Py-IR (473 K and 673 K).

2.2. Pore size distributions of Beta samples

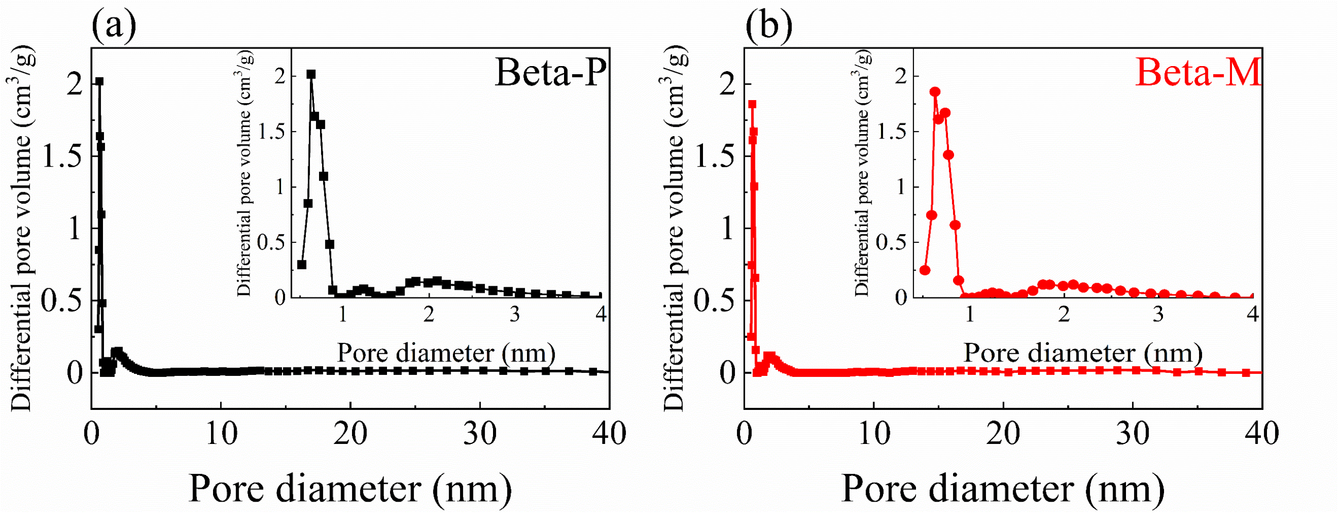

Figure S1. NLDFT pore size distributions of (a) Beta-P and (b) Beta-M.

2.3. HRTEM images and EDS-Mapping images

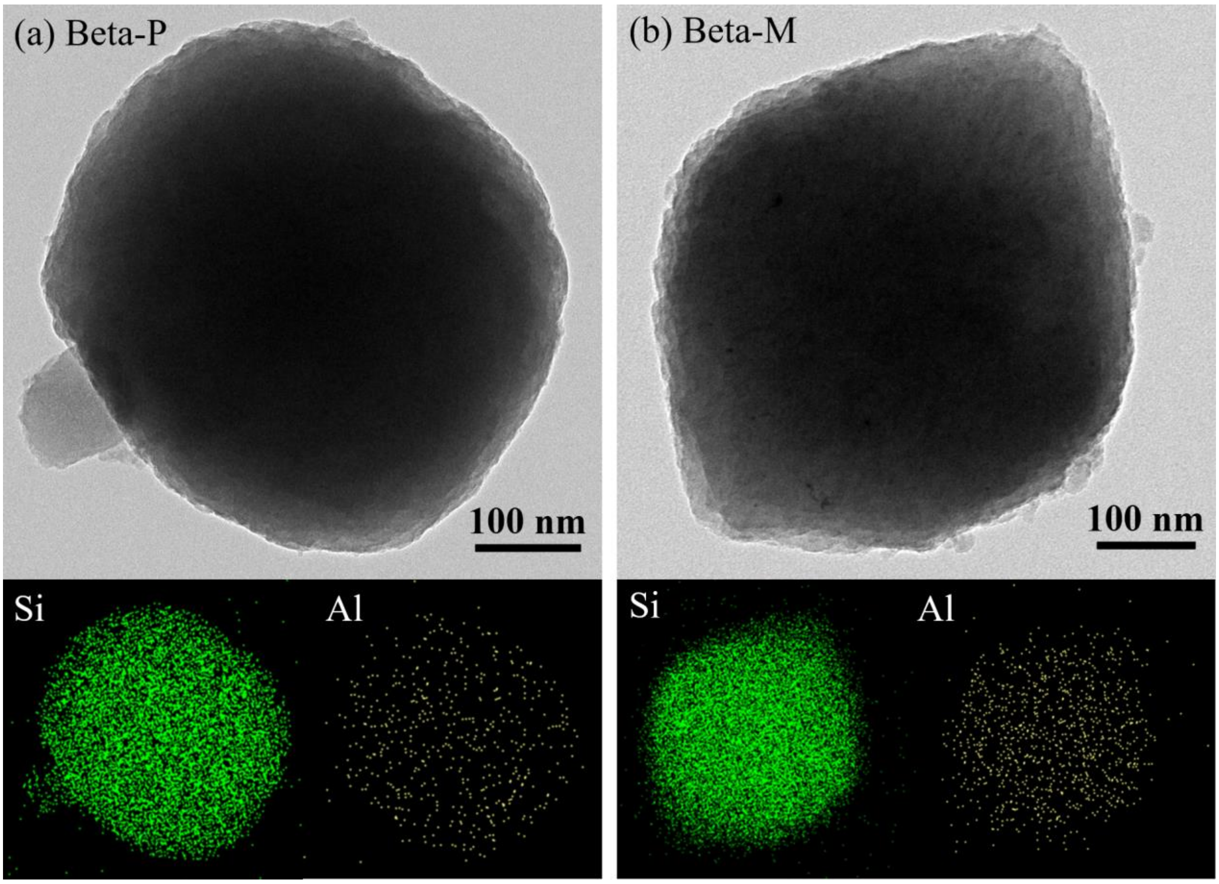

Figure S2. HRTEM images and EDS mapping images of (a) Beta-P and (b) Beta-M.

2.4. Particle size distributions of Beta samples

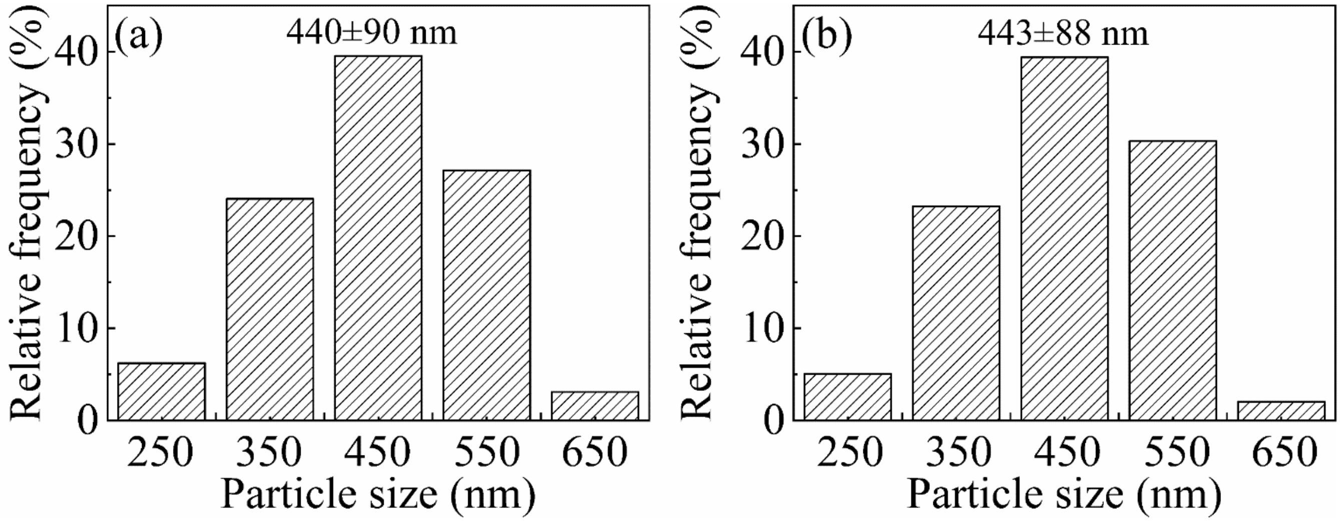

Figure S3. Particle size distributions of (a) Beta-P and (b) Beta-M.

2.5. Pt size distributions of Pt/Beta samples

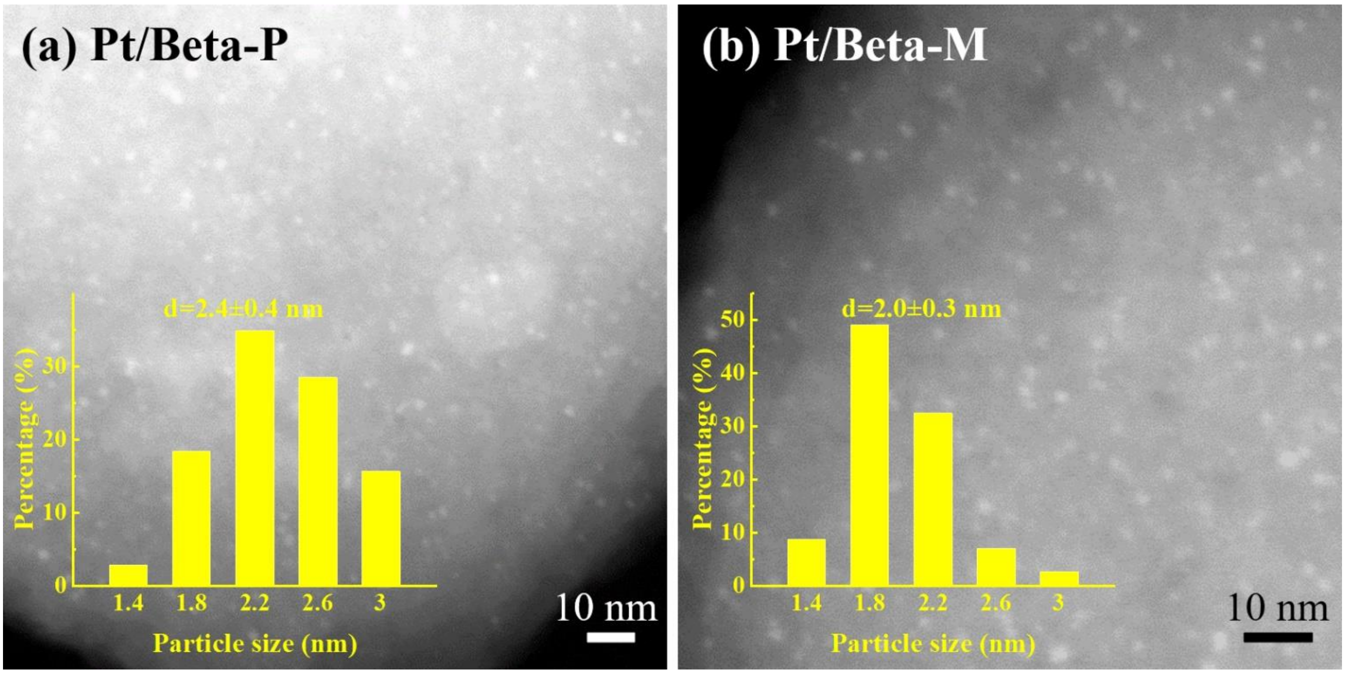

Figure S4. Representative HAADF-STEM images and Pt particle size distributions (inserted) of (a) Pt/Beta-P and (b) Pt/Beta-M.

Table S2. Characteristics of Pt particles in Pt/Beta-P and Pt/Beta-M catalysts.

Catalysts	Pt dispersion [a] (%)	Pt particle size [a] (nm)	Pt particle size [b] (nm)
Pt/Beta-P	50.5	2.2	2.4
Pt/Beta-M	54.8	2.1	2.0

[a] Pt dispersion and Pt particle size determined by CO-chemisorption
[b] Average Pt particle size determined by HAADF-STEM

2.6. Elimination of the effect of Pt

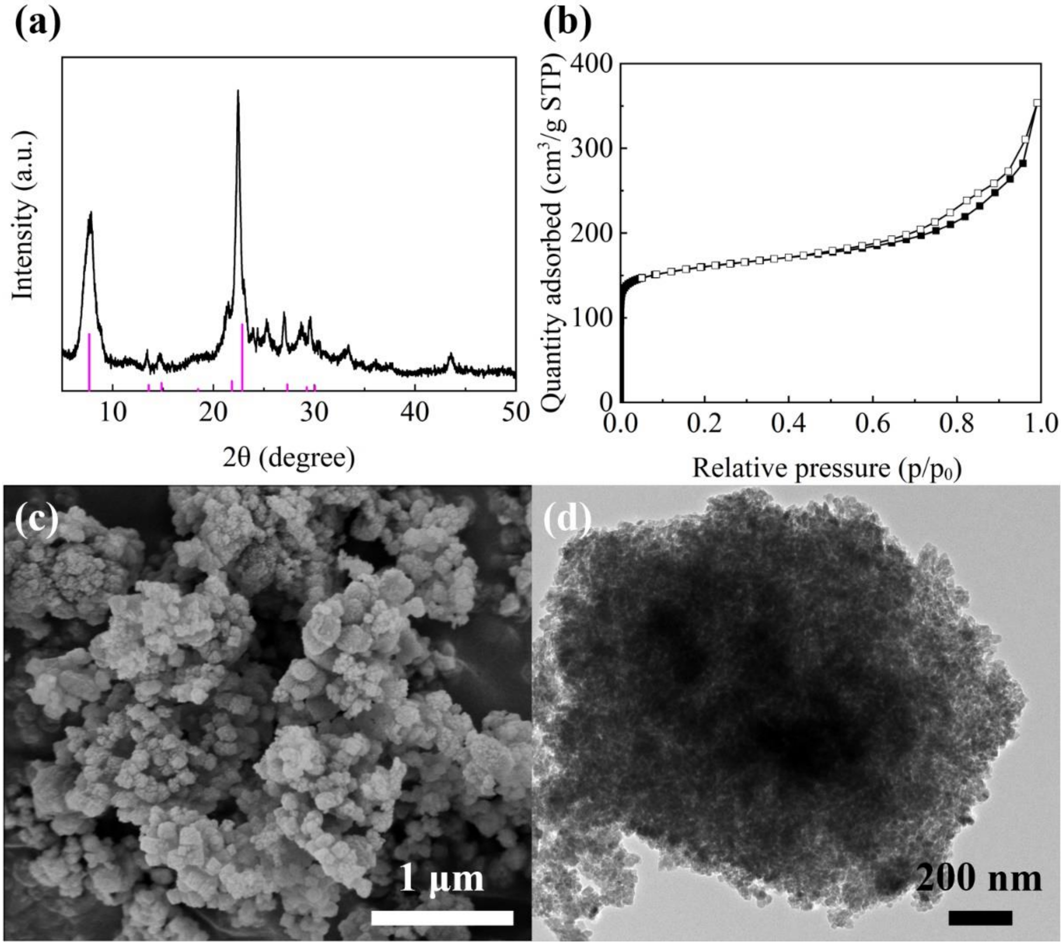

Figure S5. Structure, morphology, and texture of the commercial Beta samples. (a) Wide-angle powder XRD pattern; (b) N_2 adsorption and desorption isotherm; (c) representative SEM image and (d) representative HRTEM image.

Table S3. Characteristics of Pt particles in 0.3 wt% Pt/Beta-C and 0.5 wt% Pt/Beta-C catalysts.

Catalysts	Pt loading (%)	Pt dispersion [a] (%)	Pt particle size [a](nm)
0.3 wt% Pt/Beta-C	0.3	32.5	3.5
0.5 wt% Pt/Beta-C	0.5	33.3	3.5

[a] Pt dispersion and Pt particle size determined by CO-chemisorption

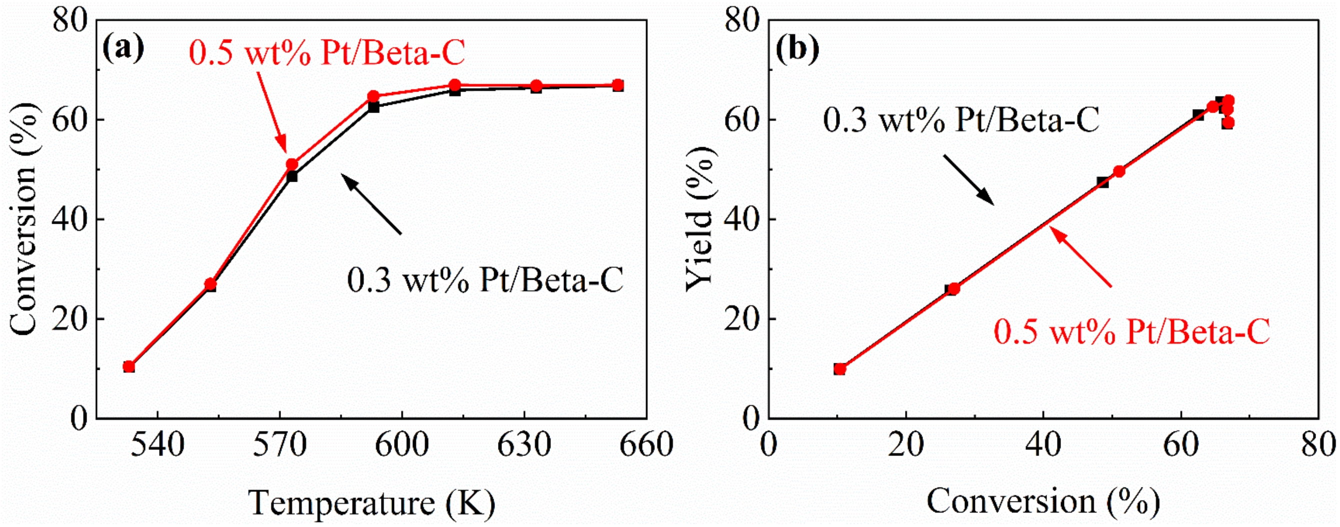

Figure S6. Catalytic performance of 0.3 wt% Pt/Beta-C and 0.5 wt% Pt/Beta-C for isomerization of *n*-pentane. (a) Conversion of *n*-pentane and (b) yield of *i*-pentane. Reaction conditions: P = 1 atm, T = 533–653 K, WHSV= 9.12 $g_{n\text{-pentane}}/(g_{Cat}\cdot h)$, H_2/*n*-pentane mole ratio = 15.8.

2.7. Effect of SiO_2 deposition on acidity

Table S4. Effect of SiO_2 deposition on the change of BAS number reported in the literature.

Zeolite type	Particle size (µm)	BAS number before SiO_2 deposition (mmol/g)	BAS number after SiO_2 deposition (mmol/g)	Reduction in BAS after SiO_2 deposition (%)	Notes	References
ZSM-5	0.3	0.86	0.69	19.8	2 cycles of deposition (using TEOS)	[8]
ZSM-5	0.3	0.86	0.46	46.5	3 cycles of deposition (using TEOS)	[8]
ZSM-5	0.3	0.61	0.51	16.4	2 cycles of deposition (using TEOS)	[8]
ZSM-5	0.3	0.61	0.22	63.9	3 cycles of deposition (using TEOS)	[8]
ZSM-5	3	0.73	0.49	32.9	2 cycles of deposition (using TEOS)	[8]
ZSM-5	3	0.73	0.33	54.8	3 cycles of deposition (using TEOS)	[8]
ZSM-5	0.5	0.213	0.187	12.2	1 cycle of deposition (using TEOS)	[9]
ZSM-5	0.5	0.213	0.154	27.7	3 cycles of deposition (using TEOS)	[9]
ZSM-5	3	0.280	0.261	6.8	1 cycle of deposition (using TEOS)	[9]
Beta	/	0.222	0.201	9.5	1 cycle of deposition (using TEOS)	[10]
Beta	/	0.222	0.179	19.4	1 cycle of deposition (using TPOS)	[10]
Beta	/	0.222	0.094	57.7	1 cycle of deposition (using TBOS)	[10]
Beta	/	0.222	0.083	62.6	1 cycle of deposition (using OMTS)	[10]

TEOS: tetra-ethoxysilane; TPOS: tetra-propoxysilane; TBOS: tetra-butoxysilane; OMTS: octamethylcyclotetrasiloxane.

2.8. ZLC results

Table S5. Apparent diffusivities and inverses of the diffusion time constants (D_{app}/R^2) of *n*-pentane in Beta-P and Beta-M at different temperatures.

Temperature (K)	Apparent diffusivity (m^2/s)		Inverse of diffusion time constant (s^{-1})	
	Beta-P	Beta-M	Beta-P	Beta-M
393	$6.75x10^{-18}$	$1.46x10^{-17}$	$1.39x10^{-4}$	$3.02x10^{-4}$
403	$7.78x10^{-18}$	$1.93x10^{-17}$	$1.61x10^{-4}$	$3.99x10^{-4}$
413	$1.01x10^{-17}$	$2.23x10^{-17}$	$2.08x10^{-4}$	$4.61x10^{-4}$
423	$1.31x10^{-17}$	$3.06x10^{-17}$	$2.71x10^{-3}$	$6.33x10^{-3}$

Table S6. Diffusion activation energies and pre-exponential factors of *n*-pentane in Beta-P and Beta-M.

Samples	E_a (kJ/mol)	D_0 (m^2/s)
Beta-P	31	$8.84x10^{-14}$
Beta-M	33	$3.23x10^{-13}$

Table S7. Surface permeabilities (α) and additional characteristic path lengths (σ) of *n*-pentane in Beta-P at different temperatures.

Temperature (K)	α (m/s)	σ (m)	σ/R
393	$3.83x10^{-9}$	$1.04x10^{-7}$	1.47
403	$3.97x10^{-9}$	$1.27x10^{-7}$	1.58
413	$5.63x10^{-9}$	$1.07x10^{-7}$	1.49
423	$6.98x10^{-9}$	$1.16x10^{-7}$	1.53

2.9. Effect of SiO_2 loading

ICP-AES results show that 5.8 wt% and 27.7 wt% of SiO_2 deposited on Beta-M and Beta-M-M, respectively. The XRD patterns (Fig. S7) show that the peak intensity at 2θ = 22.6° for Beta-M-M is lower than that of Beta-P, indicating that the crystallinity can be significantly reduced when there is a lot of SiO_2 deposited on Beta crystals. On the other hand, the peak intensity at 2θ = 22.6° for Beta-M is similar to that of Beta-P, implying that a small quantity of SiO_2 would not significantly change the crystallinity.

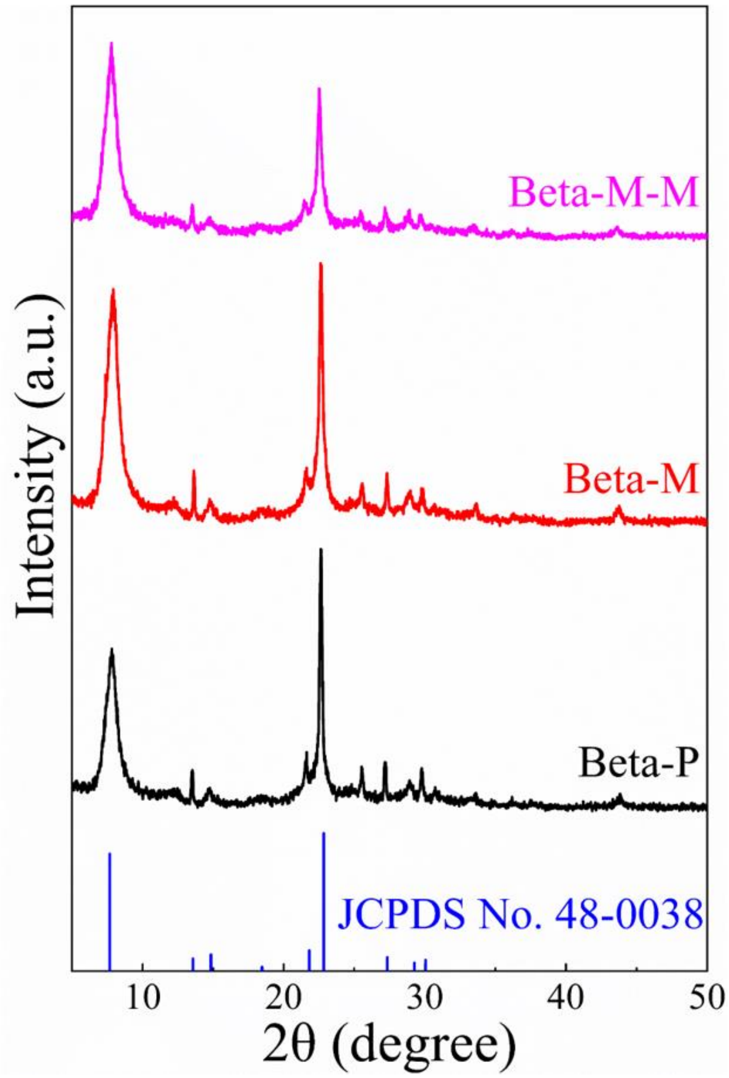

Figure S7. Wide-angle powder XRD patterns of parent and modified Beta samples.

As seen from the N_2 adsorption and desorption isotherms in Fig. S8, all the samples show a type I isotherm according to the IUPAC classification, and the deposition of SiO_2 only slightly changes the pore structure.

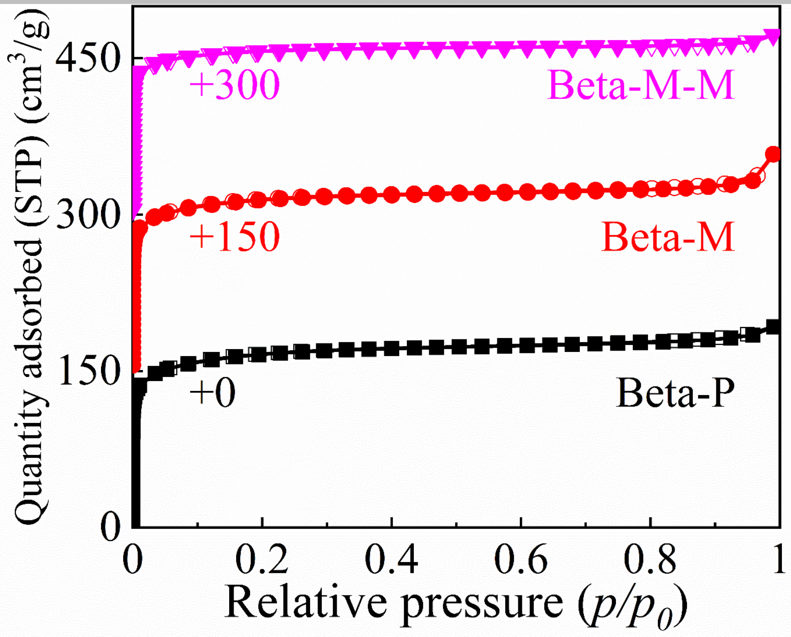

Figure S8. N_2 adsorption and desorption isotherms of parent and modified Beta samples.

The representative SEM images (Fig. S9) show that the deposition of SiO_2 does not significantly change the morphology of the samples.

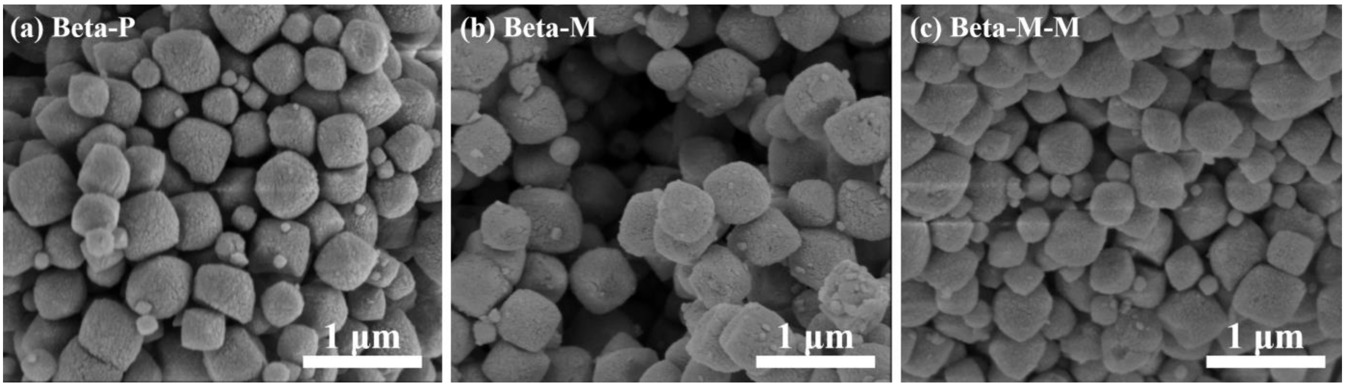

Figure S9. Representative SEM images of (a) Beta-P, (b) Beta-M, and (c) Beta-M-M.

Table. S8. Characteristics of Beta samples

Properties	Unit	Beta-P	Beta-M	Beta-M-M
S_{BET} [a]	m^2/g	514	505	478
V_{total} [b]	cm^3/g	0.30	0.32	0.27
Si/Al ratio [c]	/	25.1	26.6	32.3
Loading of SiO_2 [d]	wt%	/	5.8	27.7

[a] Specific surface area calculated by the BET method.

[b] Total volume determined from the adsorbed volume at p/p_0 = 0.99.

[c] Si/Al molar ratio measured by using ICP-AES.

[d] Loading of SiO_2 calculated by the difference in Si/Al molar ratio.

As shown in Fig. S10 and Table S9, the apparent diffusivity of *n*-pentane for Beta-M is 121-148% higher than that for Beta-P, but the apparent diffusivity for Beta-M-M is close to that for Beta-P. A proper quantity of SiO_2 can reduce surface barriers by increasing the sticking probability. However, too much SiO_2 deposited on the zeolite crystals may block many surface pores, which can enhance surface barriers. Similar results can be found in the work by Lercher and co-workers.[11,12]

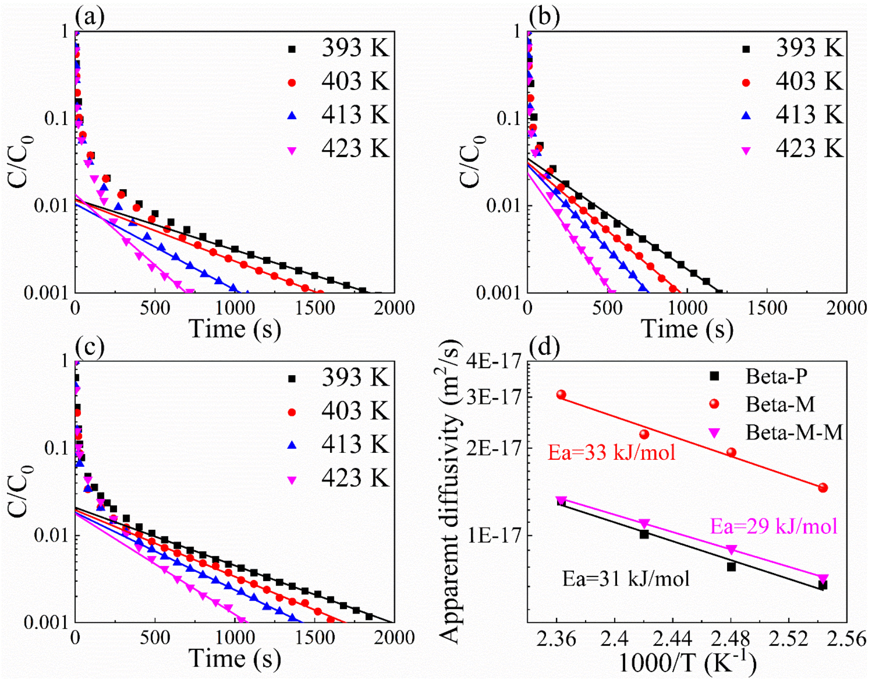

Figure S10. Apparent diffusivities of *n*-pentane measured by the zero-length column method. Desorption curves for (a) Beta-P, (b) Beta-M and (c) Beta-M-M. (C_0 and C are the initial and transient effluent

concentrations of *n*-pentane); (d) Arrhenius plots of apparent diffusivities. Measuring conditions: Flow rate = 80 mL/min, atmospheric pressure.

Table S9. Apparent diffusivities of *n*-pentane in three Beta samples.

Temperature (K)	Apparent diffusivity (m^2/s)		
	Beta-P	Beta-M	Beta-M-M
393	$6.75x10^{-18}$	$1.46x10^{-17}$	$7.13x10^{-18}$
403	$7.78x10^{-18}$	$1.93x10^{-17}$	$9.01x10^{-18}$
413	$1.01x10^{-17}$	$2.23x10^{-17}$	$1.10x10^{-17}$
423	$1.31x10^{-17}$	$3.06x10^{-17}$	$1.33x10^{-17}$

As seen from Fig. S11 and Table S10, the conversion for Pt/Beta-M is higher than for Pt/Beta-P, while the conversion for Pt/Beta-M-M is lower than for Pt/Beta-P. For example, the conversion at *t* = 1 h for Pt-Beta-M is 1.5-31.4% higher than that for Pt/Beta-P, while the conversion at *t* = 1 h for Pt-Beta-M-M is 3.8-19.3% lower than that for Pt/Beta-P. The improved catalyst performance of Pt/Beta-M can only be attributed to the reduced surface barriers after SiO_2 deposition. The reason for the decreased catalyst performance of Pt/Beta-M-M is twofold: (1) too much SiO_2 deposited on Beta crystals enhances surface barriers by blocking many surface pores; (2) SiO_2 deposition passivates the active sites for the isomerization reaction. Similar results about the decreased conversion after SiO_2 deposition can be found in the literature.[8,13–15]

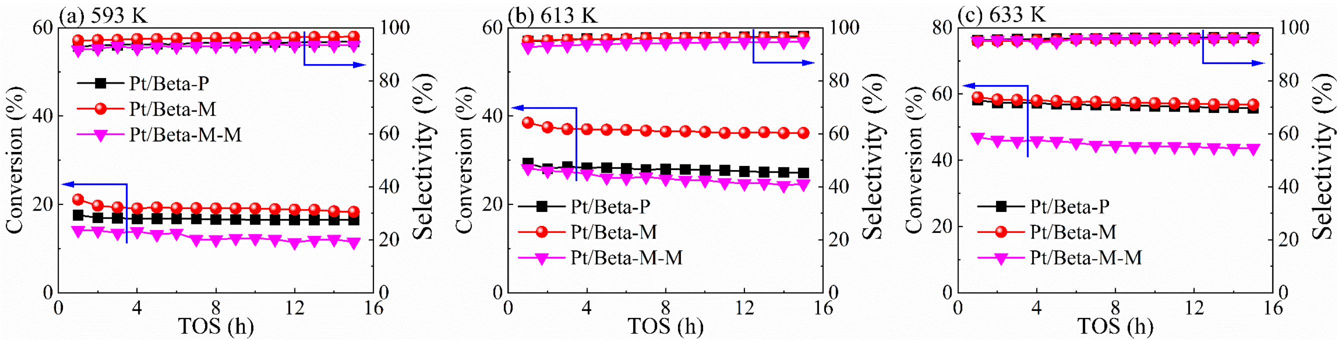

Figure S11. Catalytic performance of three Pt/Beta catalysts at (a) 593 K, (b) 613 K and (c) 633 K.

Table S10. Conversions of *n*-pentane over three Pt/Beta catalysts at different temperatures when t = 1 h.

Samples	Conversion (%)		
	593 K	613 K	633 K
Pt/Beta-P	17.6	29.3	58.1
Pt/Beta-M	21.1	38.5	59.0
Pt/Beta-M-M	14.2	28.2	47.0

Based on the above results, we infer that there exists an optimal loading of SiO_2 on Beta crystals. The optimization of SiO_2 loading is out of the scope of this work and will be the subject of future work.

References

[1] G. Ye, Y. Sun, Z. Guo, K. Zhu, H. Liu, X. Zhou, M. O. Coppens, *J. Catal.* **2018**, *360*, 152–159.

[2] S. J. Reitmeier, O. C. Gobin, A. Jentys, J. A. Lercher, *Angew. Chemie - Int. Ed.* **2009**, *48*, 533–538.

[3] C. A. Emeis, *J. Catal.* **1993**, *141*, 347–354.

[4] M. Eic, D. M. Ruthven, *Zeolites* **1988**, *8*, 40–45.

[5] M. Gao, H. Li, M. Yang, S. Gao, P. Wu, P. Tian, S. Xu, M. Ye, Z. Liu, *Commun. Chem.* **2019**, *2*, 43.

[6] S. Peng, M. Gao, H. Li, M. Yang, M. Ye, Z. Liu, *Angew. Chemie - Int. Ed.* **2020**, *59*, 21945–21948.

[7] A. R. Teixeira, C.-C. Chang, T. Coogan, R. Kendall, W. Fan, P. J. Dauenhauer, *J. Phys. Chem. C* **2013**, *117*, 25545–25555.

[8] P. Losch, M. Boltz, C. Bernardon, B. Louis, A. Palčić, V. Valtchev, *Appl. Catal. A Gen.* **2016**, *509*, 30–37.

[9] S. Zheng, H. R. Heydenrych, H. P. Röger, A. Jentys, J. A. Lercher, *Top. Catal.* **2003**, *22*, 101–106.

[10] W. O. Parker, A. De Angelis, C. Flego, R. Millini, C. Perego, S. Zanardi, *J. Phys. Chem. C* **2010**, *114*, 8459–8468.

[11] O. C. Gobin, S. J. Reitmeier, A. Jentys, J. A. Lercher, *J. Phys. Chem. C* **2011**, *115*, 1171–1179.

[12] J. A. Lercher, S. J. Reitmeier, O. C. Gobin, A. Jentys, *J. Phys. Chem. C* **2009**, *113*, 15355–15363.

[13] H. Wang, Y. Zou, *Catal. Letters* **2003**, *86*, 163–167.

[14] Y. Wang, L. Xu, Z. Yu, X. Zhang, Z. Liu, *Chinese J. Catal.* **2008**, *29*, 1237–1241.

[15] N. Chen, N. Wang, Y. Ren, H. Tominaga, E. W. Qian, *J. Catal.* **2017**, *345*, 124–134.

Author Contributions

G.Y. and M.-O.C. conceived the initial idea. S.H. carried out most of the experiments, including catalyst synthesis, characterization and tests. J.L. carried out some characterizations. X.Z. and W.Y. provided some suggestions to improve the work. G.Y., M.-O.C., and S.H. analyzed the data and wrote the manuscript.
